# Association of Longitudinal Patterns of Habitual Sleep Duration With Risk of Cardiovascular Events and All-Cause Mortality

**DOI:** 10.1001/jamanetworkopen.2020.5246

**Published:** 2020-05-22

**Authors:** Yun-He Wang, Jing Wang, Shuo-Hua Chen, Jin-Qiao Li, Qing-Dong Lu, Michael V. Vitiello, Feng Wang, Xiang-Dong Tang, Jie Shi, Lin Lu, Shou-Ling Wu, Yan-Ping Bao

**Affiliations:** 1 National Institute on Drug Dependence and Beijing Key Laboratory of Drug Dependence, Peking University, Beijing, China; 2 School of Public Health, Peking University, Beijing, China; 3 Peking University Medical Informatics Center, Peking University, Beijing, China; 4 Health Care Center, Kailuan Medical Group, Tangshan, China; 5 Department of Psychiatry and Behavioral Sciences, University of Washington, Seattle; 6 Chinese Center for Health Education, Beijing, China; 7 Sleep Medicine Center, Department of Respiratory and Critical Care Medicine, Mental Health Center and Translational Neuroscience Center, State Key Laboratory of Biotherapy, West China Hospital, Sichuan University, Chengdu, China; 8 Institute of Mental Health, National Clinical Research Center for Mental Disorders, Key Laboratory of Mental Health and Peking University Sixth Hospital, Peking University, Beijing, China; 9 Peking-Tsinghua Center for Life Sciences and International Data Group/McGovern Institute for Brain Research, Peking University, Beijing, China; 10 Department of Cardiology, Kailuan General Hospital, Tangshan, China

## Abstract

**Importance:**

Single self-reported measures of sleep duration are associated with adverse health outcomes; however, long-term patterns of self-reported sleep duration and their association with cardiovascular events (CVEs) and all-cause mortality remain unknown.

**Objective:**

To determine whether trajectories of long-term vs single-measure sleep duration are associated with subsequent risk of CVEs and all-cause mortality.

**Design, Setting, and Participants:**

The Kailuan study is a prospective, population-based cohort study that began in 2006. The present cohort included 52 599 Chinese adults without atrial fibrillation, myocardial infarction, stroke, or cancer to 2010. Trajectories in sleep duration from January 1, 2006, to December 31, 2010, were identified to investigate the association with risk of CVEs and all-cause mortality from January 1, 2010, to December 31, 2017. Data analysis was conducted from July 1 to October 31, 2019.

**Exposures:**

Habitual self-reported nocturnal sleep durations were collected in 2006, 2008, and 2010. Trajectories in sleep duration for 4 years were identified by latent mixture modeling.

**Main Outcomes and Measures:**

All-cause mortality and first incident CVEs (atrial fibrillation, myocardial infarction, and stroke) from 2010 to 2017 were confirmed by medical records. Based on the baseline sleep duration and patterns over time, 4 trajectories were categorized (normal stable, normal decreasing, low increasing, and low stable).

**Results:**

Of the 52 599 adults included in the study (mean [SD] age at baseline, 52.5 [11.8] years), 40 087 (76.2%) were male and 12 512 (23.8%) were female. Four distinct 4-year sleep duration trajectory patterns were identified: normal stable (range, 7.4 to 7.5 hours [n = 40 262]), normal decreasing (mean decrease from 7.0 to 5.5 hours [n = 8074]), low increasing (mean increase from 4.9 to 6.9 hours [n = 3384]), and low stable (range, 4.2 to 4.9 hours [n = 879]). During a mean (SD) follow-up of 6.7 (1.1) years, 2361 individuals died and 2406 had a CVE. Compared with the normal-stable pattern and adjusting for potential confounders, a low-increasing pattern was associated with increased risk of first CVEs (hazard ratio [HR], 1.22; 95% CI, 1.04-1.43), a normal-decreasing pattern was associated with increased risk of all-cause mortality (HR, 1.34; 95% CI, 1.15-1.57), and the low-stable pattern was associated with the highest risk of CVEs (HR, 1.47; 95% CI, 1.05-2.05) and death (HR, 1.50; 95% CI, 1.07-2.10).

**Conclusions and Relevance:**

In this study, sleep duration trajectories with lower or unstable patterns were significantly associated with increased risk of subsequent first CVEs and all-cause mortality. Longitudinal sleep duration patterns may assist in more precise identification of different at-risk groups for possible intervention. People reporting consistently sleeping less than 5 hours per night should be regarded as a population at higher risk for CVE and mortality.

## Key Points

**Question:**

Are longitudinal patterns of habitual sleep duration associated with subsequent risk of cardiovascular events and all-cause mortality?

**Findings:**

In this cohort study that included 52 599 participants, 4 distinct sleep duration trajectories reported during a 4-year interval were identified. Compared with a stable sleep duration of 7.0 to 8.0 hours per night, normal-decreasing and low-increasing patterns were associated with increased risk of first cardiovascular events and all-cause mortality, respectively; individuals reporting consistently sleeping less than 5.0 hours per night had the highest risk.

**Meaning:**

This study suggests that sleep duration trajectories with lower or unstable patterns may be associated with increased risk of subsequent first cardiovascular events and all-cause mortality.

## Teaser

This cohort study assesses whether longitudinal patterns of habitual sleep duration are associated with subsequent risk of cardiovascular events and all-cause mortality in adults enrolled in a population-based study in China.

## Introduction

Both short and long sleep durations are associated with an increased risk of cardiovascular events (CVEs) and all-cause mortality, as well as related risk factors such as hypertension, diabetes, and obesity.^1,2^ However, the evidence supporting these associations is based on single baseline measures of sleep durations, and the effect of longitudinal sleep duration patterns remains unknown. Nevertheless, it was reasonable to expect that habitual sleep duration might change over time, particularly in a population of older adults.^3^ In addition, despite varying by country, sleep duration shows secular trends of reduction or increase,^4^ and a single measure may bias the true association between habitual sleep duration and adverse health outcomes. A recent study^5^ found that long and unstable trajectories of weekly napping duration are related to an increased risk of obesity and hypertension, although this association was not significant in terms of mean napping duration. In addition, a laboratory study^6^ suggested that a repeating pattern of insufficient sleep may lead to long-term metabolic changes that cannot be effectively mitigated by weekend recovery sleep. These findings highlight the importance of examining the effect of long-term patterns of sleep duration beyond single or mean measures, which fail to consider the effect of change in sleep duration over time. Because sleep duration may not be a stable trait, measuring sleep duration over time would better characterize the association of interest. Moreover, few previous studies adequately account for the concurrent changes in other risk factors, such as blood pressure and body mass index, that may confound the association between sleep duration and adverse health outcomes. To our knowledge, whether trajectories of long-term vs single-measure sleep duration are associated with CVEs and all-cause mortality has not been studied in any large population.

Therefore, we investigated the association between trajectory patterns of self-reported, nocturnal sleep duration, derived from 3 repeated measures during a 4-year interval, and the risk of subsequent incident CVEs and all-cause mortality in a large prospective cohort. For comparison, we also assessed the association of a single sleep measure at baseline and cumulative mean sleep duration during the 4-year interval on the risk of developing CVEs and mortality. We hypothesized that multiple trajectories of sleep duration exist within the population and that in comparison with a pattern in which individuals report adequate duration of sleep across 4 years, extreme or unstable patterns are associated with increased risk of CVEs and all-cause mortality during a subsequent 7-year follow-up.

## Methods

### Study Population

The Kailuan study is an ongoing community-based prospective cohort study designed to investigate the risk factors for cardiac, cerebrovascular, and related diseases, as detailed elsewhere.^7,8^ Briefly, in 2006 to 2007 (referred to as the 2006 survey), 101 510 participants (81 110 men and 20 400 women) aged 18 to 98 years were recruited from the Kailuan community in Tangshan, China. All participants completed a face-to-face questionnaire survey (including demographic characteristics, medical comorbidity, medical history, medication use, and lifestyle factors, including measures of sleep), clinical examinations, and laboratory tests. Subsequently, participants were followed up biennially to update the data, and outcome events were recorded annually until death or December 31, 2017, whichever came first. In the current study, we included 57 927 individuals who participated in all 3 surveys in 2006, 2008, and 2010. We excluded participants without reported sleep duration in 2006, 2008, and 2010. We also excluded participants who died from 2006 to 2010 and those with a diagnosis of CVEs or cancer to 2010 to minimize reverse causality. Self-reported sleep duration trajectories were modeled among the remaining 52 599 participants with reports of sleep duration in surveys of 2006, 2008, and 2010 and used to assess association with incident CVEs and all-cause mortality after 2010 (a flowchart of participant inclusion appears in eFigure 1 in the Supplement).

This research was conducted according to the guidelines of the World Medical Association Declaration of Helsinki^9^ and was approved by the ethics committee of the Kailuan General Hospital. All the participants gave written informed consent. This study followed the Strengthening the Reporting of Observational Studies in Epidemiology (STROBE) reporting guideline for cohort studies.

### Assessment of Sleep Duration

Subjective, habitual, nighttime sleep duration was biennially collected during face-to-face interview by the question, “On average, how many hours of sleep have you gotten per night in the preceding 12 months?” In our main analysis, we identified sleep duration trajectories based on the repeated measurement of sleep duration in 2006, 2008, and 2010. We also examined the effects of the single measure of sleep duration in 2010 and the cumulative mean sleep duration from January 1, 2006, to December 31, 2010, on subsequent risk of CVEs and all-cause mortality from January 1, 2010, to December 31, 2017, on subsequent risk of mortality. The American Heart Association recommends 7 to 9 hours of sleep per night for cardiometabolic health.^10^ Likewise, 7 to 9 hours was considered appropriate sleep duration for adults, and 7 to 8 hours for older adults, by the National Sleep Foundation.^11^ Using the evidence-based recommendations and previous studies, we grouped the 2010 and mean sleep durations into 5 groups (<6 hours as very short sleep duration; 6 to <7 hours as short sleep duration; 7 to <8 hours as reference; 8 to <9 hours as long sleep duration; and ≥9 hours as very long sleep duration). We also collected snoring status by asking “Do you generally snore when you sleep?” with response categories of never/rarely, occasionally, and frequently at the 2010 survey.

### Assessment of CVEs and Death

The primary outcomes included all-cause mortality and first incident CVEs (fatal or nonfatal CVEs, including atrial fibrillation, myocardial infarction, and stroke). Death information was collected from the vital statistics offices. Physician-diagnosed CVEs and history of CVEs was annually collected from 4 complementary sources: (1) the Municipal Social Insurance Institution, which covered all the participants; (2) Discharge Register centers from 11 affiliated hospitals; (3) death certificates; and (4) biennial interview since 2006. All potential CVEs identified by the code from the *International Statistical Classification of Diseases and Related Health Problems, Tenth Revision*, or questionnaire were ascertained and validated by a committee of 3 cardiologists and radiologists, blind to study design. Atrial fibrillation was diagnosed based on standard 12-lead electrocardiographic findings read by specialists. Myocardial infarction was diagnosed in accordance with the World Health Organization’s Multinational Monitoring of Trends and Determinants in Cardiovascular Disease (MONICA) criteria^12^ on the basis of clinical symptoms and dynamic changes in cardiac enzyme levels and electrocardiography. Fatal cases of myocardial infarction, including sudden cardiac deaths due to cardiac arrest, were confirmed by medical record, autopsy report, or death certificate listing coronary heart disease or myocardial infarction as cause of death. Stroke was diagnosed according to the World Health Organization criteria^13^ based on symptoms and neuroimages from computed tomography or magnetic resonance imaging scans of the brain and autopsy reports. Fatal strokes were confirmed by record, cerebral autopsy, and death certificate with stroke as cause of death. Nonfatal strokes were defined as the sudden onset of focal neurological deficit with a vascular mechanism lasting more than 24 hours. In the present study, we examined 2 main subtypes of stroke: cerebral infarction and intracerebral hemorrhage (not including epidural, subdural, or subarachnoid hemorrhage).

### Assessment of Potential Covariates

Data on demographic characteristics (eg, age, sex, educational attainment, income level, occupation, family history, and marital status), medical comorbidities (eg, diabetes, hypertension, hyperlipidemia), medication use (eg, antihypertensives, hypoglycemics, and agents to lower lipid levels), and lifestyle factors (eg, smoking status, drinking status, physical activity, and habitual salt intake) were collected via questionnaire during the 2010 survey. Anthropomorphic parameters such as height, weight, and waist and hip circumference were measured by trained nurses. Blood pressure was measured 3 times in a seated position using a mercury sphygmomanometer, as detailed elsewhere.^8^ The mean value of the multiple blood pressure measures was used for analysis. Body mass index was calculated as the weight in kilograms divided by the height in square meters. Fasting blood samples (>12 hours) were collected and analyzed in the laboratory of Kailuan General Hospital to obtain biochemical parameters, including levels of fasting blood glucose, high-sensitivity C-reactive protein, and creatine. Estimated glomerular filtration rate was calculated using the Chronic Kidney Disease Epidemiology Collaboration creatine equation.^14^

### Statistical Analysis

Data analysis was conducted from July 1 to October 31, 2019. Latent mixture models were fit to identify subgroups that share the similar underlying trajectories of sleep duration.^15^ We estimated multiple trajectories using the censored normal model, which is appropriate for continuous outcomes. We tested models with varied numbers and forms (eg, intercept, linear, or quadratic slope) of potential patterns. Model fit was assessed using the Bayesian information criterion. We initiated models with 5 classes and all trajectory classes in quadratic form, then compared the Bayesian information criterion with models with 4, 3, 2, and 1 classes. The models with 4 classes identified fit best, and we then compared the Bayesian information criterion of models with different functional forms. Finally, the model had 3 classes with linear order terms and 1 class with up to a quadratic order term. We also calculated the mean posterior estimated probability of final group membership to test discrimination. Person-years for each participant were calculated from the completion date of the 2010 survey until date of incident CVEs, death, loss to follow-up (1778 of 52 599 [3.4%]), or December 31, 2017, whichever occurred first. Cox proportional hazards regression models were used to estimate the association between exposures (eg, sleep duration trajectories, single measure in 2010, and cumulative mean during 2006-2010) and the risk of incident CVEs and all-cause mortality. The adjusted models included baseline sleep duration in 2010, age, sex, marital status, occupation, income level, educational attainment, smoking and drinking status, salt intake, family history of stroke and myocardial infarction, diabetes (defined as a self-reported physician-diagnosed history, currently taking hypoglycemic medication, or a fasting blood glucose concentration ≥126 mg/dL [to convert to millimoles per liter, multiply by 0.0555]), hypertension (defined as a self-reported physician-diagnosed history, systolic blood pressure ≥140 mm Hg or diastolic blood pressure ≥90 mm Hg, or currently using antihypertensives), snoring status, and mean body mass index, systolic and diastolic blood pressure, estimated glomerular filtration rate, fasting blood glucose level, and high-sensitivity C-reactive protein level during 2006 to 2010. Potential confounders were selected based on a priori knowledge of factors associated with sleep duration and the risk of death and CVEs. Proportional hazards assumptions were assessed with tests based on Schoenfeld residuals and log-log inspection, and no variables violated the assumption.

Stratified analyses by sex, age (<65 vs ≥65 years), hypertension (yes vs no), diabetes (yes vs no), kidney function (estimated glomerular filtration rate <60 vs ≥60 mL/min/1.73 m^2^), and body mass index (calculated as weight in kilograms divided by height in meters squared; <25.0 vs ≥25.0 kg/m^2^) were performed to examine potential statistical interaction. Given the concern of reverse causality, we reexamined the effect of sleep duration trajectories after excluding participants who developed CVEs or died in the first 2 years of follow-up. In addition, to correct the effect of sleep apnea, we performed sensitivity analyses by exclusion of those with self-reported snoring. Considering the effects of shift work, we also removed potential shift workers. To rule out the effect of weight change and fatigue, we further excluded those who developed cancer during follow-up. Because atrial fibrillation is not generally considered a CVE, we reanalyzed the data without including atrial fibrillation in outcomes. We also adjusted for sleep duration in 2006 to examine whether the association between sleep duration trajectories and CVEs or mortality was attributed to the single measure of sleep duration. All analyses were performed using SAS software, version 9.4 (SAS Institute, Inc), and 2-sided *P* < .05 indicated significance.

## Results

During the 3-survey rounds in 2006, 2008, and 2010, 101 510 individuals participated in at least 1 survey, 86 615 in more than 1 survey, and 57 927 in all 3 surveys. This last group formed the study sample. We excluded 2978 participants without reported sleep duration, 885 who died during 2006 to 2010, and 1465 participants with a diagnosis of CVE or cancer to 2010. Those participants who were excluded owing to missing sleep duration data were older (mean [SD] age, 57.7 [12.0] vs 49.0 [11.8] years; *P* < .001) and more likely to be female (724 [24.3%] vs 12 571 [23.9%]; *P* < .001) but had similar future risk of CVEs (age- and sex-adjusted hazard ratio [HR], 0.95; 95% CI, 0.81-1.12) and mortality (age- and sex-adjusted HR, 1.09; 95% CI, 0.95-1.25), relative to those with 3 completed sleep duration data sets. Of the 52 599 participants finally included in the study (mean [SD] age at baseline, 52.5 [11.8] years), 40 087 (76.2%) were male and 12 512 (23.8%) were female. Four discrete trajectories in sleep duration during the 4-year span were identified (Figure): 40 262 participants (76.5%) maintained normal sleep duration throughout (normal-stable group; mean range, 7.4 to 7.5 hours); 879 (1.7%) had relatively short sleep duration throughout (low-stable group; mean range, 4.2 to 4.9 hours); 8074 (15.4%) started with normal levels and experienced a decrease in sleep duration (normal-decreasing group; mean sleep duration decrease, 7.0 to 5.5 hours); and 3384 (6.4%) started with short sleep duration and experienced an increase in sleep duration (low-increasing group; mean sleep duration increase, 4.9 to 6.9 hours). Mean (SD) probabilities for each individual being in the final group membership ranged from 0.70 (0.15) to 0.91 (0.13) across the trajectory groups. The basic characteristics of participants in 2010 by sleep duration trajectories are shown in Table 1.

**Figure.**
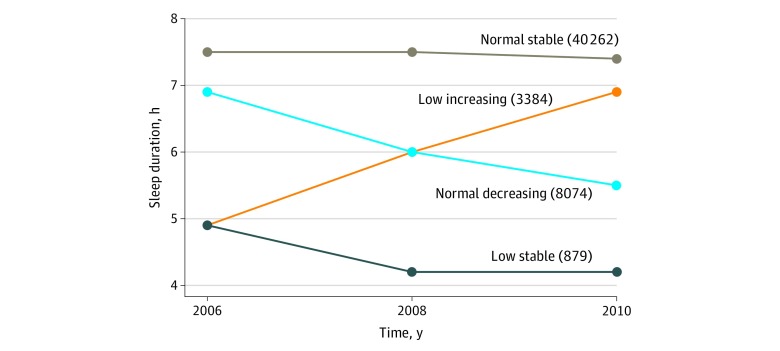
Sleep Duration Trajectories During 2006 to 2010 in Kailuan Study The normal-stable sleep duration pattern ranged from 7.4 to 7.5 hours per night; low-increasing pattern, mean increase from 4.9 to 6.9 hours per night; normal-decreasing pattern, mean decrease from 7.0 to 5.5 hours per night; and low-stable pattern, range of 4.9 to 4.2 hours per night.

**Table 1.** Characteristics of the Participants by Sleep Duration Trajectory Group

Characteristics	Sleep duration trajectory^a^			
	Normal		Low	
	Stable	Decreasing	Increasing	Stable
All participants	40 262 (76.5)	8047 (15.3)	3384 (6.5)	879 (1.7)
Age, mean (SD), y	52.0 (11.9)	53.5 (11.3)	55.8 (11.6)	57.1 (11.9)
Age ≥65 y	5595 (13.9)	1273 (15.8)	734 (21.7)	231 (26.3)
Female	9872 (24.5)	1740 (21.6)	665 (19.7)	235 (26.7)
Unmarried	220 (0.5)	33 (0.4)	13 (0.4)	3 (0.3)
Mean income ≥3000 RMB/mo^b^	4514 (11.7)	901 (11.3)	297 (8.9)	63 (7.2)
Blue collar worker^b^	32 837 (84.7)	6518 (83.4)	2880 (88.5)	720 (84.2)
Educational attainment college or university	4921 (12.2)	957 (11.9)	259 (7.7)	72 (8.2)
Current smoker	12 927 (32.1)	3452 (42.9)	1254 (37.1)	344 (39.1)
Current alcohol user	13 275 (33.0)	3646 (45.3)	1374 (40.6)	398 (45.3)
Salt intake ≥10 g/d	3705 (9.2)	1196 (14.9)	359 (10.6)	147 (16.7)
Physical activity ≥3 times/wk	5205 (12.9)	1492 (18.5)	587 (17.3)	191 (21.7)
Hypertension	5175 (12.9)	1684 (20.9)	684 (20.2)	247 (28.1)
Diabetes	1505 (3.7)	484 (6.0)	194 (5.7)	73 (8.3)
Hyperlipidemia	1880 (4.7)	672 (8.4)	215 (6.4)	95 (10.8)
Frequent snoring	4772 (11.9)	1734 (21.5)	574 (17.0)	188 (21.4)
Medication use^a^				
Antihypertensive	3674 (9.2)	1194 (14.9)	499 (14.9)	189 (21.7)
Hypoglycemic	1089 (2.7)	358 (4.5)	145 (4.3)	55 (6.3)
Agents to lower lipid levels	306 (0.8)	107 (1.3)	29 (0.9)	12 (1.4)
Family history				
Stroke	882 (2.2)	331 (4.1)	77 (2.3)	47 (5.3)
MI	421 (1.0)	163 (2.0)	45 (1.3)	19 (2.2)
BMI, mean (SD)^c^	24.9 (3.2)	25.0 (3.1)	25.1 (3.2)	25.0 (3.3)
hs-CRP level, mg/dL^c^^,^^d^	1.6	1.6	1.7	1.5
Blood pressure, mm Hg, mean (SD)^c^				
SBP	129.0 (16.9)	129.0 (16.3)	131.9 (17.2)	131.4 (17.1)
DBP	83.5 (9.3)	83.4 (8.9)	84.3 (9.1)	83.8 (9.2)
FBG level, mean (SD), mg/dL^c^	99.1 (25.2)	99.1 (23.4)	100.9 (27.0)	99.1 (25.2)
eGFR, mean (SD), mL/min/1.73 m^2^	87.6 (18.5)	91.7 (16.6)	88.9 (17.7)	90.3 (16.6)
Sleep duration, mean (SD), h				
Year 2006	7.5 (0.8)	6.9 (0.9)	4.9 (0.9)	4.9 (1.3)
Year 2008	7.5 (0.9)	6.0 (1.0)	6.0 (1.2)	4.2 (1.5)
Year 2010	7.4 (0.8)	5.5 (0.8)	6.9 (0.9)	4.2 (1.4)

Abbreviations: BMI, body mass index (calculated as weight in kilograms divided by height in square meters); DBP, diastolic blood pressure; eGFR, estimated glomerular filtration rate; FBG, fasting blood glucose; hs-CRP, high-sensitivity C-reactive protein; MI, myocardial infarction; RMB, renminbi; SBP, systolic blood pressure.

SI conversion factors: To convert CRP to mg/L, multiply by 10; glucose to mmol/L, multiply by 0.0555.

^a^ Unless otherwise indicated, data are expressed as number (percentage) of patients. The normal-stable sleep duration pattern ranged from 7.4 to 7.5 hours per night; low-increasing pattern, mean increase from 4.9 to 6.9 hours per night; normal-decreasing pattern, mean decrease from 7.0 to 5.5 hours per night; and low-stable pattern, range of 4.9 to 4.2 hours per night.

^b^ Variable had missing value. Data were missing for income for 947 participants (1.8%), occupation for 1894 (3.6%), use of antihypertensives for 421 (0.8%), and use of hypoglycemics for 210 (0.4%).

^c^ Mean data based on measures in 2006, 2008, and 2010.

^d^ Indicates geometric mean.

During 352 413 person-years of follow-up (mean [SD] follow-up, 6.7 [1.1] years), a total of 4418 participants died (n = 2361) or had a CVE (n = 2406). Sleep duration trajectories were significantly associated with the risk of CVEs and all-cause mortality (Table 2). Compared with the normal-stable group, who maintained a sleep duration of 7.0 to 8.0 hours per night for 4 years, low-stable and low-increasing patterns were significantly associated with higher risk of first CVEs after adjustment for potential confounders. Adjusted HRs of CVEs were 1.22 (95% CI, 1.04-1.43) for the low-increasing pattern, 1.13 (95% CI, 0.97-1.32) for the normal-decreasing pattern, and 1.47 (95% CI, 1.05-2.05) for the low-stable pattern. Relative to the normal-stable group, risk of all-cause mortality was significantly higher in those with normal-decreasing and low-stable sleep duration pattern. Adjusted HRs of death were 1.34 (95% CI, 1.15-1.57) for the normal-decreasing pattern, 0.95 (95% CI, 0.80-1.13) for the low-increasing pattern, and 1.50 (95% CI, 1.07-2.10) for the low-stable pattern. Sensitivity analysis excluding outcome events that occurred in the first 2 years of follow-up, shift workers, those who developed cancers during follow-up, those with self-reported frequent snoring, or atrial fibrillation from outcomes indicated consistent results (Table 3). In addition, further adjustment for sleep duration in 2006 did not materially alter our results. We also performed stratified analysis by age, sex, and baseline comorbidities (Table 4 and eTables 1 and 2 in the Supplement). No significant interaction was observed for any of the medical comorbidities, and the results were similar when stratified by baseline weight status and sex. However, when stratified by age group, the association with CVEs was found for the low-stable (HR, 1.75; 95% CI, 1.17-1.62) and low-increasing (HR, 1.28; 95% CI, 1.04-1.56) groups among participants younger than 65 years, but not among those 65 years or older.

**Table 2.** Association of Sleep Duration Trajectories During 2006 to 2010 With Cardiovascular Events and All-Cause Mortality

Outcome	Sleep duration trajectory^a^			
	Normal		Low	
	Stable	Decreasing	Increasing	Stable
**Cardiovascular events and all-cause mortality**				
No. of cases/person-years	3299/271 626	695/54 608	332/22 635	92/5849
Incidence rate^b^	1214.5	1272.7	1422.6	1572.9
Age- and sex-adjusted HR (95% CI)	1 [Reference]	1.01 (0.93-1.10)	1.03 (0.92-1.16)	1.09 (0.89-1.34)
Multivariable-adjusted HR (95% CI)^c^	1 [Reference]	1.25 (1.11-1.40)	1.07 (0.95-1.21)	1.48 (1.16-1.89)
**Cardiovascular events**				
No. of cases/person-years	1793/271 945	366/54 656	197/22 658	50/5851
Incidence rate^b^	659.3	669.6	869.4	854.5
Age- and sex-adjusted HR (95% CI)	1 [Reference]	0.99 (0.88-1.10)	1.17 (1.01-1.36)	1.15 (0.87-1.53)
Multivariable-adjusted HR (95% CI)^c^	1 [Reference]	1.13 (0.97-1.32)	1.22 (1.04-1.43)	1.47 (1.05-2.05)
**All-cause mortality**				
No. of cases/person years	1769/276 896	380/55 649	163/23 204	49/5984
Incidence rate^b^	638.9	682.8	702.4	818.8
Age- and sex-adjusted HR (95% CI)	1 [Reference]	1.03 (0.92-1.15)	0.89 (0.76-1.05)	1.02 (0.76-1.35)
Multivariable-adjusted HR (95% CI)^c^	1 [Reference]	1.34 (1.15-1.57)	0.95 (0.80-1.13)	1.50 (1.07-2.10)

Abbreviation: HR, hazard ratio.

^a^ The normal-stable sleep duration pattern ranged from 7.4 to 7.5 hours per night; low-increasing pattern, mean increase from 4.9 to 6.9 hours per night; normal-decreasing pattern, mean decrease from 7.0 to 5.5 hours per night; and low-stable pattern, range of 4.9 to 4.2 hours per night.

^b^ Indicates per 100 000 person-years.

^c^ Adjusted for age, sex, marital status (single, married, or divorced), occupation (blue collar or white collar), mean income (<50, 500-3000, or ≥3000 renminbi/mo), educational attainment (illiteracy or elementary, middle school, or college/university), physical activity (never, 1-2 times/wk, or ≥3 times/wk), smoking status (never, past, or current), alcohol consumption status (never, past, or current), salt intake (<6, 6-10, or >10 g/d), family history of stroke (yes or no), family history of myocardial infarction (yes or no), hypertension (yes or no), hyperlipidemia (yes or no), diabetes (yes or no), snoring frequency (never/rare, occasionally, or frequently), sleep duration in 2010, antihypertensive use (yes or no), hypoglycemic use (yes or no), use of agents to lower lipid levels (yes or no), body mass index (calculated as weight in kilograms divided by height in square meters; <18.5, 18.5 to <25.0, 25.0 to <30.0, or ≥30.0), fasting blood glucose level (<72, 72 to <101, 101 to <110, 110 to <126, or ≥126 mg/dL [to convert to mmol/L, multiply by 0.0555]), high-sensitivity C-reactive protein (<0.10, 0.10 to <0.30, 0.30 to <1.0, or ≥1.0 mg/dL [to convert to mg/L, multiply by 10]), systolic blood pressure (<120, 120 to <140, or ≥140 mm Hg), diastolic blood pressure (<80, 80 to <90, or ≥90 mm Hg), and estimated glomerular filtration rate (<30, 30 to <60, 60 to <90, or ≥90 mL/min/1.73 m^2^).

**Table 3.** Sensitivity Analyses on Associations Between Sleep Duration Trajectory Group and the First CVE and All-Cause Mortality

Outcomes	Sleep duration trajectory, HR (95% CI)^a^			
	Normal		Low	
	Stable	Decreasing	Increasing	Stable
**SA1: Exclusion of participants who had events (CVEs or death) in the first 2 y of follow-up (n = 51 679)**				
CVEs	1 [Reference]	1.16 (0.97-1.38)	1.23(1.02-1.47)	1.39 (0.93-2.07)
Death	1 [Reference]	1.44 (1.21-1.72)	0.97 (0.81-1.18)	1.58 (1.08-2.32)
Total	1 [Reference]	1.29 (1.14-1.47)	1.07 (0.93-1.23)	1.42 (1.07-1.89)
**SA2: Further adjusted for sleep duration in 2006 based on fully adjusted model**				
CVEs	1 [Reference]	1.12 (0.96-1.32)	1.19 (1.01-1.44)	1.42 (1.00-2.03)
Death	1 [Reference]	1.35 (1.15-1.58)	0.98 (0.80-1.20)	1.55 (1.08-2.21)
Total	1 [Reference]	1.25 (1.11-1.40)	1.08 (0.93-1.25)	1.49 (1.15-1.93)
**SA3: Exclusion of shift workers (n = 50 559)**^**b**^				
CVEs	1 [Reference]	1.12 (0.96-1.31)	1.21 (1.03-1.42)	1.44 (1.03-2.02)
Death	1 [Reference]	1.34 (1.15-1.56)	0.95 (0.80-1.13)	1.48 (1.06-2.08)
Total	1 [Reference]	1.24 (1.10-1.39)	1.06 (0.94-1.20)	1.46 (1.14-1.86)
**SA4: Exclusion of participants who developed cancers during follow-up (n = 52 425)**				
CVEs	1 [Reference]	1.13 (0.96-1.32)	1.22 (1.05-1.43)	1.44 (1.03-2.02)
Death	1 [Reference]	1.35 (1.16-1.58)	0.95 (0.80-1.13)	1.21 (1.07-2.11)
Total	1 [Reference]	1.25 (1.11-1.40)	1.07 (0.95-1.21)	1.47 (1.15-1.88)
**SA5: Exclusion of those with self-reported frequent snoring (n = 45 331)**				
CVEs	1 [Reference]	1.18 (0.98-1.41)	1.19 (0.99-1.43)	1.56 (1.05-2.30)
Death	1 [Reference]	1.45 (1.21-1.73)	0.99 (0.82-1.19)	1.77 (1.21-2.58)
Total	1 [Reference]	1.34 (1.18-1.53)	1.06 (0.93-1.22)	1.72 (1.30-2.27)
**SA6: Exclusion of atrial fibrillation from outcomes (n = 52 850)**				
CVEs	1 [Reference]	1.12 (0.95-1.31)	1.22 (1.04-1.44)	1.54 (1.09-2.17)
Death	1 [Reference]	1.34 (1.15-1.56)	0.94 (0.79-1.11)	1.55 (1.12-2.14)
Total	1 [Reference]	1.24 (1.11-1.40)	1.08 (0.95-1.22)	1.54 (1.21-1.97)

Abbreviations: CVE, cardiovascular event; HR, hazard ratio; SA, sensitivity analysis.

^a^ The normal-stable sleep duration pattern ranged from 7.4 to 7.5 hours per night; low-increasing pattern, mean increase from 4.9 to 6.9 hours per night; normal-decreasing pattern, mean decrease from 7.0 to 5.5 hours per night; and low-stable pattern, range of 4.9 to 4.2 hours per night. Models were adjusted for age, sex, marital status (single, married, or divorced), occupation (blue collar or white collar), mean income (<50, 500-3000, or ≥3000 renminbi/mo), educational attainment (illiteracy or elementary, middle school, or college/university), physical activity (never, 1-2 times/wk, or ≥3 times/wk), smoking status (never, past, or current), alcohol consumption status (never, past, or current), salt intake (<6, 6-10, or >10 g/d), family history of stroke (yes or no), family history of myocardial infarction (yes or no), hypertension (yes or no), hyperlipidemia (yes or no), diabetes (yes or no), snoring frequency (never/rare, occasionally, or frequently), sleep duration in 2010, antihypertensive use (yes or no), hypoglycemic use (yes or no), use of agents to lower lipid levels (yes or no), body mass index (calculated as weight in kilograms divided by height in square meters; <18.5, 18.5 to <25.0, 25.0 to <30.0, or ≥30.0), fasting blood glucose level (<72, 72 to <101, 101 to <110, 110 to <126, or ≥126 mg/dL [to convert to mmol/L, multiply by 0.0555]), high-sensitivity C-reactive protein (<.10, .10 to <.30, .30 to <1.0, or ≥1.0 mg/dL [to convert to mg/L, multiply by 10]), systolic blood pressure (<120, 120 to <140, or ≥140 mm Hg), diastolic blood pressure (<80, 80 to <90, or ≥90 mm Hg), and estimated glomerular filtration rate (<30, 30 to <60, 60 to <90, or ≥90 mL/min/1.73 m^2^).

^b^ Shift workers were self-reported in the 2016 survey.

**Table 4.** Association Between Sleep Duration Trajectory Groups and the Composite of First CVEs and All-Cause Mortality, Stratified by Age, Sex, and Baseline Comorbidities

Variable	Sleep duration trajectory group^a^				*P* value for interaction
	Normal		Low		
	Stable	Decreasing	Increasing	Stable	
**Age, y**					
<65 (n = 44 766)					.46
Incidence rate^b^	1967/237 614	416/46 802	170/18 133	47/4425	
Fully adjusted model HR (95% CI)^c^	1 [Reference]	1.22 (1.05-1.41)	1.12 (0.95-1.33)	1.60 (1.15-2.24)	
≥65 (n = 7833)					
Incidence rate^b^	1332/34 332	279/7854	162/4525	45/1423	
Fully adjusted model HR (95% CI)^c^	1 [Reference]	1.28 (1.06-1.53)	1.01 (0.85-1.21)	1.39 (0.97-2.00)	
**Sex**					
Male (n = 40 087)					.65
Incidence rate^b^	293/204 580	615/42 876	291/18 220	69/4318	
Fully adjusted model HR (95% CI)^c^	1 [Reference]	1.24 (1.10-1.41)	1.06 (0.93-1.21)	1.38 (1.04-1.82)	
Female (n = 12 512)					
Incidence rate^b^	366/67 357	80/11 780	41/4438	23/1533	
Fully adjusted model HR (95% CI)^c^	1 [Reference]	1.23 (0.87-1.74)	1.07 (0.76-1.52)	2.03 (1.14-3.61)	
**BMI**					
<25.0 (n = 27 346)					.68
Incidence rate^b^	1513/142 634	339/28 259	143/11 331	48/3206	
Fully adjusted model HR (95% CI)^c^	1 [Reference]	1.32 (1.11-1.56)	0.99 (0.83-1.19)	1.49 (1.05-2.12)	
≥25.0 (n = 25253)					
Incidence rate^b^	1786/129 293	356/26 393	189/11 321	44/2645	
Fully adjusted model HR (95% CI)^c^	1 [Reference]	1.18 (1.01-1.39)	1.13 (0.96-1.33)	1.50 (1.06-2.12)	
**Hypertension**					
Yes (n = 7790)					.20
Incidence rate^b^	732/33 668	224/11 047	113/4405	38/1602	
Fully adjusted model HR (95% CI)^c^	1 [Reference]	1.07 (0.87-1.32)	1.12 (0.91-1.39)	1.35 (0.91-2.01)	
No (n = 44 775)					
Incidence rate^b^	2567/238 106	470/43 564	219/18 226	54/4243	
Fully adjusted model HR (95% CI)^c^	1 [Reference]	1.35 (1.17-1.54)	1.04 (0.90-1.21)	1.58 (1.15-2.16)	
**Diabetes **					
Yes (n = 2256)					.15
Incidence rate^b^	250/9692	86/3141	29/1249	19/447	
Fully adjusted model HR (95% CI)^c^	1 [Reference]	1.16 (0.81-1.67)	0.87 (0.56-1.36)	2.23 (1.16-4.29)	
No (n = 50 336)					
Incidence rate^b^	3049/262 202	609/51 498	303/21 401	73/5404	
Fully adjusted model HR (95% CI)^c^	1 [Reference]	1.26 (1.11-1.42)	1.09 (0.97-1.24)	1.37 (1.05-1.79)	
**Hyperlipidemia**					
Yes (n = 2862)					.22
Incidence rate^b^	158/12 634	76/4469	20/1453	13/610	
Fully adjusted model HR (95% CI)^c^	1 [Reference]	1.25 (1.11-1.41)	1.08 (0.96-1.23)	1.51 (1.16-1.96)	
No (n = 49 736)					
Incidence rate^b^	3141/259 263	619/50 185	312/21 200	79/5240	
Fully adjusted model HR (95% CI)^c^	1 [Reference]	1.17 (0.79-1.72)	0.89 (0.50-1.40)	1.10 (0.52-2.34)	
**Kidney function**					
eGFR<60 mL/min/1.73 m^2^ (n = 3314)					.60
Incidence rate^b^	462/18 037	51/1904	41/1334	9/226	
Fully adjusted model HR (95% CI)^c^	1 [Reference]	1.94 (1.12-3.3 6)	1.15 (0.74-1.77)	4.17 (1.60-10.83)	
eGFR≥60 mL/min/1.73 m^2^ (n = 49 285)					
Incidence rate^b^	2837/253 787	644/52 748	291/21 323	83/5624	
Fully adjusted model HR (95% CI)^c^	1 [Reference]	1.22 (1.08-1.37)	1.05 (0.92-1.19)	1.41 (1.09-1.82)	

Abbreviations: BMI, body mass index (calculated as weight in kilograms divided by height in square meters); CVE, cardiovascular event; eGFR, estimated glomerular filtration rate; HR, hazard ratio.

^a^ The normal-stable sleep duration pattern ranged from 7.4 to 7.5 hours per night; low-increasing pattern, mean increase from 4.9 to 6.9 hours per night; normal-decreasing pattern, mean decrease from 7.0 to 5.5 hours per night; and low-stable pattern, range of 4.9 to 4.2 hours per night.

^b^ Indicates per 100 000 person-years.

^c^ Adjusted for age, sex, marital status (single, married, or divorced), occupation (blue collar or white collar), mean income (<50, 500-3000, or ≥3000 renminbi/mo), educational attainment (illiteracy or elementary, middle school, or college/university), physical activity (never, 1-2 times/wk, or ≥3 times/wk), smoking status (never, past, or current), alcohol consumption status (never, past, or current), salt intake (<6, 6-10, or >10 g/d), family history of stroke (yes or no), family history of myocardial infarction (yes or no), hypertension (yes or no), hyperlipidemia (yes or no), diabetes (yes or no), snoring frequency (never/rare, occasionally, or frequently), sleep duration in 2010, antihypertensive use (yes or no), hypoglycemic use (yes or no), use of agents to lower lipid levels (yes or no), body mass index (calculated as weight in kilograms divided by height in square meters; <18.5, 18.5 to <25.0, 25.0 to <30.0, or ≥30.0), fasting blood glucose level (<72, 72 to <101, 101 to <110, 110 to <126, or ≥126 mg/dL [to convert to mmol/L, multiply by 0.0555]), high-sensitivity C-reactive protein (<.10, .10 to <.30, .30 to <1.0, or ≥1.0 mg/dL [to convert to mg/L, multiply by 10]), systolic blood pressure (<120, 120 to <140, or ≥140 mm Hg), diastolic blood pressure (<80, 80 to <90, or ≥90 mm Hg), and estimated glomerular filtration rate (<30, 30 to <60, 60 to <90, or ≥90 mL/min/1.73 m^2^).

A U-shaped association of single sleep duration in 2010 with CVEs and death is shown in eFigure 2 in the Supplement. Participants with sleep duration of 7.0 to 8.0 hours per night had the lowest risk of all outcomes. After adjustment for potential confounders, short and long sleep durations were associated with CVEs and death. Compared with sleeping 7.0 to less than 8.0 hours per night, adjusted HRs for the composite outcomes were 1.24 (95% CI, 1.10-1.39) for those who slept less than 6.0 hours per night, 1.08 (95% CI, 0.98-1.20) for those who slept 6.0 to less than 7.0 hours per night, 1.32 (95% CI, 1.21-1.44) for those who slept 8.0 to less than 9.0 hours per night, and 1.45 (95% CI, 1.13-1.87) for those who slept at least 9.0 hours per night. The results were similar for CVEs and all-cause mortality individually (eTable 3 in the Supplement). In contrast, mean sleep duration during 2006 to 2010 was not associated with subsequent risk of CVEs. Longer sleep duration (HR for 8.0 to <9.0 hours, 1.11 [95% CI, 1.00-1.23]; HR for ≥9 hours per night, 2.34 [95% CI, 1.46-3.72]) but not shorter sleep duration (HR for <6 hours per night, 0.98 [95% CI, 0.84-1.14]; HR for 6 to <7 hours per night, 0.99 [95% CI, 0.89-1.09]) was associated with the risk of all-cause mortality (eTable 4 in the Supplement).

## Discussion

The present study provides new findings suggesting that trajectories in sleep duration were significantly associated with the risk of the first CVEs and death, even after adjustment for a single measure of baseline sleep duration, and supports conventional evidence that single measures of sleep duration were associated with adverse health outcomes. Four heterogeneous trajectories in sleep duration during a 4-year span were identified, and these patterns were associated with subsequent risk of death and CVEs. Compared with the normal-stable duration trajectory, which is consistent with a normal sleep pattern, low-increasing and low-stable duration trajectories were associated with increased risk for CVEs, whereas low-stable and normal-decreasing trajectories were associated with increased risk for all-cause mortality. Participants who exhibited a low-stable sleep pattern, maintaining a nocturnal sleep duration of less than 5.0 hours during the 4-year assessment period, had the highest risk of death and CVEs. A U-shaped association between single measures of sleep duration at baseline and future adverse events was also observed. Participants with short (<6.0 hours per night) and long (8.0 to <9.0 hours per night) sleep duration had increased risk of adverse health outcomes, regardless of their earlier sleep patterns. The findings suggest that trajectories of long-term sleep duration are associated with subsequent risk of CVEs and death besides one-off measures closer to the time of events.

This study is, to our knowledge, the first to investigate the association of longitudinal patterns of sleep duration with CVEs and all-cause mortality in a large prospective cohort. Previous studies of the association between sleep duration and health typically measured sleep duration at 1 point, few studies examined the effect of sleep duration change at 2 points, and none to our knowledge considered the pattern of sleep duration during a prolonged period. Our results are supported by a previous study showing that participants who experience a decrease or an increase in sleep duration measured at 2 phases had a higher risk of all-cause mortality.^16^ The present study extends those findings to demonstrate that not only is change of sleep duration across 2 points important, but certain sleep duration trajectories were associated with CVEs and death. A decreasing sleep pattern may represent a progressive curtailment of sleep duration, facilitated by sleep disorders originating from psychosocial causes and medical comorbidities. However, the association with death was not significantly altered after adjustment for sociodemographic variables, snoring frequency, and existing medical morbidity. Individuals who experienced an increasing sleep pattern may represent those who initially had inadequate sleep duration and then slept longer to compensate the sleep debt. A recent study^6^ found that “sleeping in” cannot mitigate metabolic disruptions linked to sleep deficit and may even make them worse. This finding suggests that sleep deprivation has long-term adverse consequences, which may not be ameliorated by sleep compensation. In addition, an increasing duration sleep pattern may be simply a marker of subclinical disease, and the observed association with CVEs may be due to reverse causality. However, further excluding participants who had events within the first 2 years did not alter the result.

We observed that a short-stable sleep pattern was associated with the highest risk of all-cause mortality and CVEs. Similarly, findings from a working cohort^17^ showed that prolonged inadequate sleep duration (defined as sleep of <7.0 h/d in 2 phases 4 to 7 years apart) is associated with all-cause death during a 25-year period. Participants who experienced the short-stable pattern may represent those with chronic sleep deprivation, including spontaneous short sleepers who stay up late and compulsory short sleepers, such as shift workers or those with sleep problems. Further excluding potential shift workers and those with self-reported frequent snoring resulted in similar findings, suggesting that long-term lack of sleep, per se, may have pernicious effects on health. People reporting consistently sleeping 5.0 hours or less per night may be regarded as a higher-risk population for CVE and mortality.

Some mechanisms may contribute to the association between chronic sleep curtailment and the risk of CVEs and death. Evidence suggests that sleep debt has an adverse effect on carbohydrate metabolism and levels of endocrine hormones such as insulin, cortisol, and leptin, which may contribute to the alterations of appetite and glucose metabolism and accelerate the development of obesity and diabetes.^18,19^ Sleep restriction in adult men with normal habitual sleep patterns resulted in increased activity of the sympathetic nervous system, serum norepinephrine, and proinflammatory cytokines (interleukin 1β, interleukin 6, and C-reactive protein), which were independently associated with cardiovascular diseases and death.^20,21^ The mechanisms underlying the association between sleep extension and adverse outcomes are considered more speculative. Increased sleep duration may represent a marker of undiagnosed diseases or the effect of uncontrolled comorbidity, such as obstructive sleep apnea, leading to the risk of mortality and CVEs. In addition, those who initially had inadequate sleep duration may have begun to sleep longer to compensate for the sleep debt and may have developed an increased duration pattern. Evidence also suggests that those with a subjective long sleep pattern may have poor sleep efficiency, which was associated with increased risk of death.^22^ Further investigation is warranted to examine the potential association of such increased sleep duration with health outcomes.

The findings from the present study provide unique insight into the association of long-term patterns of sleep duration with CVEs and death. These heterogeneous trajectories may be useful to distinguish individuals at risk more accurately than single or mean measures of sleep duration. For example, although the association between short single-measure sleep duration and the risk of CVEs in our study replicated findings from previous evidence,^23,24^ we found the low-increasing pattern but not the normal-decreasing pattern was associated with the risk of CVEs. Moreover, those participants with patterns of short to increasing sleep still tended to have increased risk despite apparently improving their sleep duration, indicating the lack of sleep may have long-term adverse consequences and the importance of stable adequate sleep. In contrast, shorter mean sleep duration during the 4-year interval was not associated with the risk of all-cause mortality. Charting the trajectories of sleep duration in association with health outcomes may reveal additional information that cannot be captured by single or mean measurement. The better understanding of the effect and timing of change in sleep duration may help to identify populations with higher risk who can then be targeted with interventions to promote cardiovascular health and healthy sleep.

### Strengths and Limitations

The strengths of the present study include using longitudinal assessment of habitual sleep duration to examine the association of long-term sleep patterns with CVEs and mortality in a large community-based population. We also adjusted for mean body mass index, blood pressure, concentrations of fasting blood glucose and high-sensitivity C-reactive protein and estimated glomerular filtration rate based on 3 measures of these biomarkers across 4 years, as well as other cardiovascular risk factors, which may greatly reduce unmeasured residual confounding.

Several limitations of this study are worth noting. First, our cohort only included Chinese adults from the Kailuan community, and most were male; therefore, findings may not be generalizable to other populations. However, similar associations between habitual sleep duration and the risk of CVEs and mortality have been observed in individuals from multiple geographic regions with different cultural backgrounds and income levels, suggesting the broad, generalizable nature of the data.^23,25^ Second, potential bias might occur by using self-reported sleep duration as a substitute for objective measures of sleep duration. However, the use of objective methods (eg, polysomnography) may not be feasible in large studies of a general population. Previous studies have shown that subjective estimates of sleep duration and sleep parameters monitored by actigraphy and polysomnography are highly correlated.^26,27^ Importantly, sleep patterns were identified based on repeated measures over time, which may reduce misclassification. Third, the lack of other dimensions of sleep, such as sleep disorders (eg, sleep apnea and insomnia), daytime nap duration, and sleep quality, further limit investigation. We attempted to correct for sleep disorders by exclusion of individuals with frequent snoring and shift workers, which left the results unchanged. Daytime nap duration, as a compensation for sleep debt during the night or a habitual behavior, may further influence the risk of CVEs in individuals with specific nocturnal sleep duration.^23^ Although sleep quality also has been associated with the risk of adverse outcomes,^10,24^ previous studies^28^ reported a strong association of a sleep duration of 7 to 8 hours with better sleep quality, and we intended to focus on the duration of sleep because it is more likely to be a modifiable factor than sleep quality.^28^ Further studies with detailed sleep parameters are needed to replicate our findings. In addition, despite statistical adjustment for multiple potential covariates, residual confounding and reverse causality cannot be ruled out, and results should be interpreted with caution.

## Conclusions

Altogether, the findings of this study provide new insights for the association of habitual sleep duration with CVEs and mortality, highlighting the importance of the temporal rather than static behavior of sleep duration. Sleep duration trajectories with lower or unstable patterns were significantly associated with increased risk of subsequent first CVEs and all-cause mortality. Additional studies are warranted to confirm the utility of specific sleep duration trajectories in risk prediction and to explore the effect of lifestyle modification and intervention on sleep trajectories and associated outcomes in routine clinical practice and public health settings.

## Notes

Supplement. **eFigure 1.** Flowchart of Participant Inclusion **eFigure 2.** Restricted Spline Curves for Associations Between Baseline (2010) Sleep Duration and All-Cause Mortality or Cardiovascular Events **eTable 1. **Association Between Sleep Duration Trajectory Groups and First Cardiovascular Events, Stratified by Age, Sex, BMI, Hypertension, Diabetes, Hyperlipidemia, and Kidney Function **eTable 2. **Association Between Sleep Duration Trajectory Groups and All-Cause Mortality, Stratified by Age, Sex, BMI, Hypertension, Diabetes, Hyperlipidemia, and Kidney Function **eTable 3. **Association of Baseline (2010) Sleep Duration and First Cardiovascular Events or All-Cause Mortality **eTable 4. **Association Between Cumulative Average Sleep Duration During 2006 to 2010 and First Cardiovascular Events or All-Cause Mortality
