# Supplementary material for: Association of Longitudinal Patterns of Habitual Sleep Duration With Risk of Cardiovascular Events and All-Cause Mortality
Source: JAMA Netw Open. 2020 May 22;3(5):e205246. doi: 10.1001/jamanetworkopen.2020.5246 (PMC7244989; doi:10.1001/jamanetworkopen.2020.5246)
Supplement: Supplement. — eFigure 1. Flowchart of Participant Inclusion eFigure 2. Restricted Spline Curves for Associations Between Baseline (2010) Sleep Duration and All-Cause Mortality or Cardiovascular Events eTable 1. Association Between Sleep Duration Trajectory Groups and First Cardiovascular Events, Stratified by Age, Sex, BMI, Hypertension, Diabetes, Hyperlipidemia, and Kidney Function eTable 2. Association Between Sleep Duration Trajectory Groups and All-Cause Mortality, Stratified by Age, Sex, BMI, Hypertension, Diabetes, Hyperlipidemia, and Kidney Function eTable 3. Association of Baseline (2010) Sleep Duration and First Cardiovascular Events or All-Cause Mortality eTable 4. Association Between Cumulative Average Sleep Duration During 2006 to 2010 and First Cardiovascular Events or All-Cause Mortality [file jamanetwopen-3-e205246-s001.pdf]

Supplementary Online Content

Wang Y-H, Wang J, Chen S-H, et al. Association of longitudinal patterns of habitual sleep duration with risk of cardiovascular events and all-cause mortality. *JAMA Netw Open*. 2020;3(5):e205246. doi:10.1001/jamanetworkopen.2020.5246

eFigure 1. Flowchart of Participant Inclusion

eFigure 2. Restricted Spline Curves for Associations Between Baseline (2010) Sleep Duration and All-Cause Mortality or Cardiovascular Events

eTable 1. Association Between Sleep Duration Trajectory Groups and First Cardiovascular Events, Stratified by Age, Sex, BMI, Hypertension, Diabetes, Hyperlipidemia, and Kidney Function

eTable 2. Association Between Sleep Duration Trajectory Groups and All-Cause Mortality, Stratified by Age, Sex, BMI, Hypertension, Diabetes, Hyperlipidemia, and Kidney Function

eTable 3. Association of Baseline (2010) Sleep Duration and First Cardiovascular Events or All-Cause Mortality

eTable 4. Association Between Cumulative Average Sleep Duration During 2006 to 2010 and First Cardiovascular Events or All-Cause Mortality

This supplementary material has been provided by the authors to give readers additional information about their work.

©2020 Wang Y-H et al. *JAMA Network Open*.

eFigure 1. Flowchart of Participant Inclusion

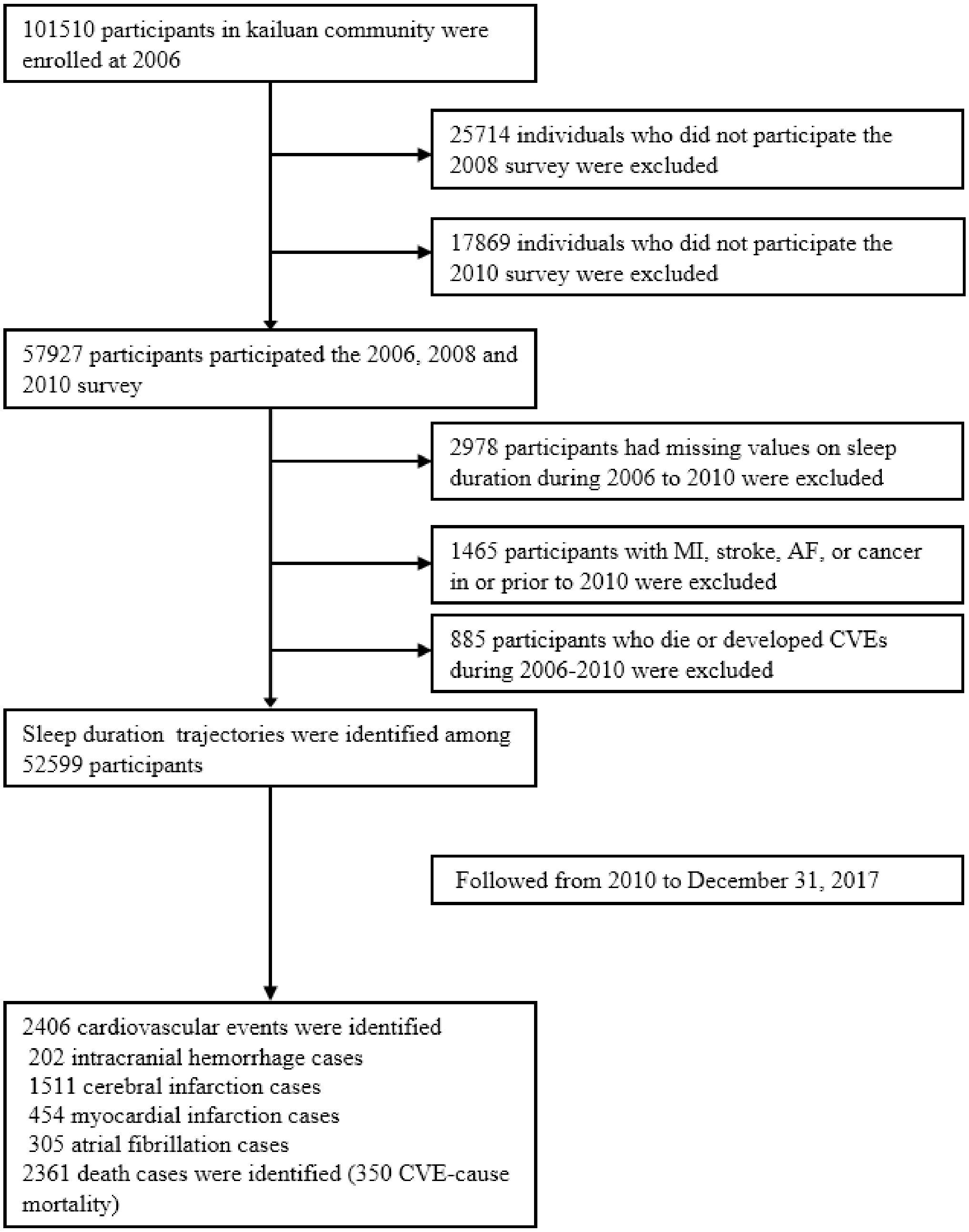

©2020 Wang Y-H et al. *JAMA Network Open*.

eFigure 2. Restricted Spline Curves for Associations Between Baseline (2010) Sleep Duration and All-Cause Mortality or Cardiovascular Events

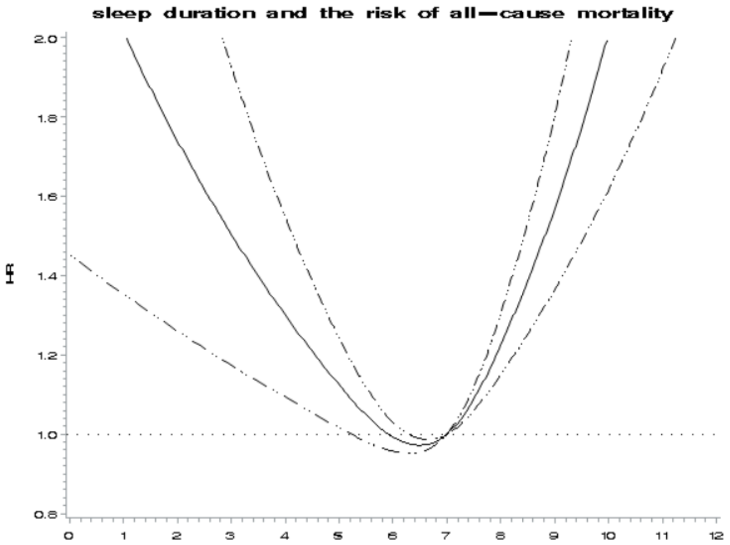

(A)

(B)

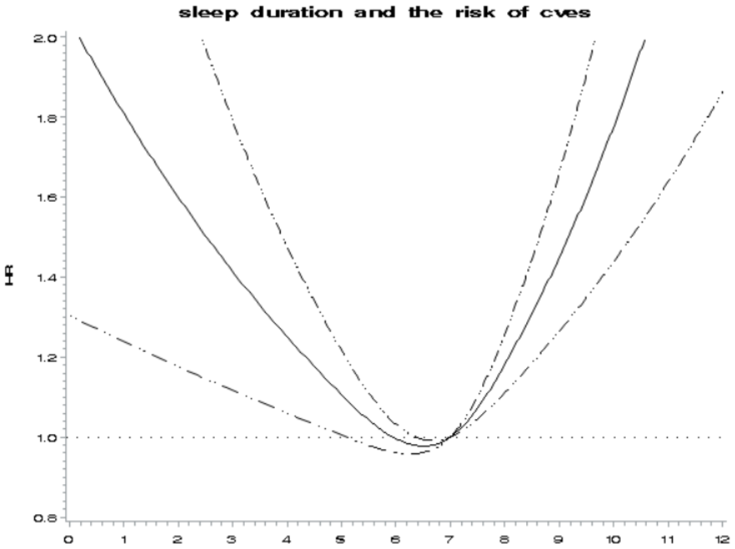

©2020 Wang Y-H et al. *JAMA Network Open*.

(C)

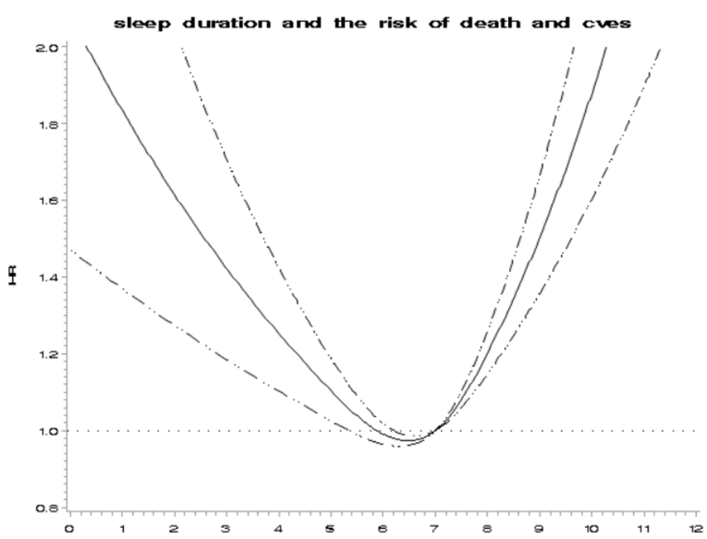

(A) all-cause mortality; (B) cardiovascular events; (C) composite outcomes; Adjusted for adjusted for age, sex, marital status, occupation, average income, education, physical activity, smoking status, drinking status, salt intake, family history of stroke, family history of MI, hypertension, hyperlipidemia, diabetes, snoring frequence, antihypertensive use, hypoglycemic use, lipid lowing agents use, BMI, fasting blood glucose, hs-CRP, SBP, DBP, eGFR.

©2020 Wang Y-H et al. *JAMA Network Open*.

eTable 1. Association Between Sleep Duration Trajectory Groups and First Cardiovascular Events, Stratified by Age, Sex, BMI, Hypertension, Diabetes, Hyperlipidemia, and Kidney Function

	Sleep duration trajectory group, HR (95% CI)				P for interaction
	**Normal-Stable**	**Normal-Decreasing**	**low-Increasing**	**low-Stable**	
**Age (years)**					0.13
<65 years (n=44766)					
Incidence rate†	1201/223292	253/43926	116/16993	35/4139	
Fully adjusted model*	1.00 (Ref)	1.13 (0.94-1.36)	**1.28 (1.04-1.56)**	**1.75 (1.17-2.62)**	
≥65 years (n=7833)					
Incidence rate†	592/32277	113/7346	81/4213	15/1320	
Fully adjusted model*	1.00 (Ref)	1.14 (0.89-1.48)	1.15 (0.89-1.48)	1.12 (0.61-2.02)	
**Sex**					
Male (n=40087)					0.78
Incidence rate†	1576/192486	325/40265	169/17073	39/4032	
Fully adjusted model*	1.00 (Ref)	1.15 (0.97-1.35)	**1.20 (1.01-1.42)**	1.42 (0.98-2.06)	
Female (n=12512)					
Incidence rate†	217/63087	41/11007	28/4134	11/1427	
Fully adjusted model*	1.00 (Ref)	1.05 (0.66-1.67)	1.37 (0.89-2.09)	1.90 (0.86-4.18)	
**BMI**					
<25 kg/m^2 (n=27346)					0.28
Incidence rate†	746/134087	167/26534	69/10607	23/2992	
Fully adjusted model*	1.00 (Ref)	1.14 (0.90-1.45)	1.02 (0.75-1.33)	1.27 (0.76-2.10)	
≥25 kg/m^2 (n=25253)					
Incidence rate†	1047/121487	199/24737	128/10599	27/2467	
Fully adjusted model*	1.00 (Ref)	1.11 (0.90-1.36)	**1.39 (1.14-1.69)**	**1.64 (1.05-2.56)**	
**Hypertension**					
Yes (n=7790)					0.78
Incidence rate†	451/31573	132/10359	71/4121	21/1496	
Fully adjusted model*	1.00 (Ref)	1.00 (0.77-1.31)	1.20 (0.91-1.57)	1.24 (0.73-2.09)	
No (n=44775)					
Incidence rate†	1342/223847	233/44138	126/17064	29/3957	
Fully adjusted model*	1.00 (Ref)	1.22 (1.00-1.48)	**1.23 (1.01-1.50)**	**1.73 (1.12-2.68)**	
**Diabetes mellitus**					
Yes (n=2256)					0.49
Incidence rate†	134/9102	40/2948	15/1288	10/420	
Fully adjusted model*	1.00 (Ref)	0.87 (0.60-1.26)	0.89 (0.52-1.53)	1.43 (0.71-2.85)	
No (n=50336)					
Incidence rate†	1659/246447	326/48309	182/20039	40/5039	
Fully adjusted model*	1.00 (Ref)	1.15 (0.97-1.35)	**1.24 (1.05-1.47)**	1.38 (0.96-1.98)	
**Hyperlipidemia**					
Yes (n=2862)					0.17
Incidence rate†	88/11824	45/4182	17/1359	8/566	
Fully adjusted model*	1.00 (Ref)	**1.50 (1.04-2.17)**	1.57 (0.92-2.70)	1.38 (0.62-3.05)	
No (n=49736)					
Incidence rate	1705/243743	321/47089	180/19847	42/4893	
Fully adjusted model	1.00 (Ref)	1.12 (0.95-1.32)	**1.22 (1.03-1.44)**	**1.48 (1.04-2.12)**	
**Kidney function**					
eGFR <60 mL/min/1.73m^2 (n=3314)					0.61
Incidence rate†	201/17114	24/1797	19/1262	2/211	
Fully adjusted model*	1.00 (Ref)	1.67 (0.82-3.42)	1.52 (0.91-2.54)	1.95 (0.40-9.48)	
eGFR ≥60 mL/min/1.73m^2 (n=49285)					
Incidence rate†	1589/238460	342/49473	178/19944	48/5248	
Fully adjusted model*	1.00 (Ref)	1.09 (0.93-1.28)	**1.20 (1.02-1.42)**	**1.42 (1.01-2.00)**	

*Adjusted for Age, sex, marital status (single, married, or divorced), occupation (Blue collar/white collar), average income (<500, 500-3,000, or ≥3,000 RMB/month), education (illiteracy or elementary, middle school, or college/university), physical activity (never, 1-2 times/week, or ≥3 times/week), smoking status (never, past, or current), drinking status (never, past, or current), salt intake (<6, 6-10, >10g/d), family history of stroke (yes/no), family history of MI (yes/no), hypertension (yes/no), hyperlipidemia (yes/no), diabetes (yes/no), snoring frequency (never/rare, occasionally, or frequently), sleep duration in 2010, antihypertensive use (yes/no), hypoglycemic use (yes/no), lipid lowing agents use (yes/no), BMI (<18.5,18.5-25.0, 25.0-30.0, or ≥30.0 kg/m^2), fasting blood glucose (<4.0, 4.0-5.60, 5.60-6.10, 6.10-7.0, or ≥7.0 mmol/L), hs-CRP (<1.0, 1.0-3.0, 3.0-10.0, or ≥10.0 mg/L), SBP (<120, 120-140, ≥140mmHg), DBP (<80, 80-90, ≥90mmHg), eGFR (<30, 30-60, 60-90, or ≥90 ml/min/1.73m^2).
CI, confidence interval; CVEs, cardiovascular events; HR, hazard ratio.
†Per 10^5 person-years.

©2020 Wang Y-H et al. *JAMA Network Open*.

eTable 2. Association Between Sleep Duration Trajectory Groups and All-Cause Mortality, Stratified by Age, Sex, BMI, Hypertension, Diabetes, Hyperlipidemia, and Kidney Function

	Sleep duration trajectory group, HR (95% CI)				P for interaction
	Normal-Stable	Normal-Decreasing	low-Increasing	low-Stable	
**Age (years)**					0.76
<65 years (n=44766)					
Incidence rate†	871/240683	182/47447	60/18439	15/4538	
Fully adjusted model*	1.00 (Ref)	**1.33 (1.07-1.66)**	0.90 (0.68-1.19)	1.35 (0.77-2.38)	
≥65 years (n=7833)					
Incidence rate†	898/35895	198/8154	103/4742	34/1471	
Fully adjusted model*	1.00 (Ref)	**1.32 (1.05-1.65)**	0.97 (0.78-1.21)	**1.52 (1.00-2.34)**	
**Sex**					
Male (n=40087)					0.51
Incidence rate†	1592/208703	335/43721	148/18671	36/4428	
Fully adjusted model*	1.00 (Ref)	**1.32 (1.12-1.56)**	0.97 (0.81-1.16)	1.39 (0.95-2.03)	
Female (n=12512)					
Incidence rate†	176/67875	45/11879	15/4510	13/1580	
Fully adjusted model*	1.00 (Ref)	1.48 (0.92-2.39)	0.78 (0.44-1.36)	2.10 (0.93-4.73)	
**BMI**					
<25 kg/m^2 (n=27346)					0.84
Incidence rate†	898/144390	201/28663	88/11503	29/3276	
Fully adjusted model*	1.00 (Ref)	**1.48 (1.19-1.84)**	1.01 (0.80-1.28)	**1.72 (1.10-2.69)**	
≥25 kg/m^2 (n=25253)					
Incidence rate†	871/132188	179/26938	75/11678	20/2732	
Fully adjusted model*	1.00 (Ref)	1.22 (0.97-1.53)	0.88 (0.68-1.13)	1.30 (0.77-2.19)	
**Hypertension**					
Yes (n=7790)					0.31
Incidence rate†	354/34847	114/11420	52/4590	22/1663	
Fully adjusted model*	1.00 (Ref)	1.18 (0.88-1.58)	1.01 (0.73-1.38)	1.63 (0.94-2.82)	
No (n=44775)					
Incidence rate†	1415/241565	266/44138	111/18570	27/4339	
Fully adjusted model*	1.00 (Ref)	**1.44 (1.20-1.73)**	0.93 (0.76-1.14)	1.41 (0.91-2.19)	
**Diabetes mellitus**					
Yes (n=2256)					0.64
Incidence rate†	148/10021	52/3234	15/1288	10/474	
Fully adjusted model*	1.00 (Ref)	1.24 (0.76-2.01)	0.63 (0.61-1.25)	2.32 (0.96-5.58)	
No (n=50336)					
Incidence rate†	1621/266528	328/52352	148/21887	39/5534	
Fully adjusted model*	1.00 (Ref)	**1.36 (1.16-1.61)**	0.99 (0.83-1.19)	1.36 (0.94-1.98)	
**Hyperlipidemia**					
Yes (n=2862)					0.21
Incidence rate†	76/12883	36/4586	4/1550	7/632	
Fully adjusted model*	1.00 (Ref)	1.31 (0.73-2.32)	0.30 (0.09-0.97)	1.24 (0.42-3.71)	
No (n=49736)					
Incidence rate	1693/263687	344/51015	159/21681	42/5376	
Fully adjusted model	1.00 (Ref)	**1.34 (1.14-1.58)**	0.99 (0.83-1.18)	**1.50 (1.04-2.13)**	
**Kidney function**					
eGFR <60 mL/min/1.73m^2 (n=3314)					0.24
Incidence rate†	306/18576	31/1963	26/1385	8/229	
Fully adjusted model*	1.00 (Ref)	**1.94 (1.12-3.36)**	1.15 (0.74-1.77)	**4.17 (1.60-10.83)**	
eGFR ≥60 mL/min/1.73m^2 (n=49285)					
Incidence rate†	1589/238460	342/49473	178/19944	48/5248	
Fully adjusted model*	1.00 (Ref)	1.09 (0.93-1.28)	**1.20 (1.02-1.42)**	**1.42 (1.01-2.00)**	

*Adjusted for Age, sex, marital status (single, married, or divorced), occupation (Blue collar/white collar), average income (<500, 500-3,000, or ≥3,000 RMB/month), education (illiteracy or elementary, middle school, or college/university), physical activity (never, 1-2 times/week, or ≥3 times/week), smoking status (never, past, or current), drinking status (never, past, or current), salt intake (<6, 6-10, >10g/d), family history of stroke (yes/no), family history of MI (yes/no),hypertension (yes/no), Hyperlipidemia (yes/no), diabetes (yes/no), snoring frequency (never/rare, occasionally, or frequently), sleep duration in 2010, antihypertensive use (yes/no), hypoglycemic use (yes/no), lipid lowing agents use (yes/no), BMI (<18.5,18.5-25.0, 25.0-30.0, or ≥30.0 kg/m^2), fasting blood glucose (<4.0, 4.0-5.60, 5.60-6.10, 6.10-7.0, or ≥7.0 mmol/L), hs-CRP (<1.0, 1.0-3.0, 3.0-10.0, or ≥10.0 mg/L), SBP (<120, 120-140, ≥140mmHg), DBP (<80, 80-90, ≥90mmHg), eGFR (<30, 30-60, 60-90, or ≥90 ml/min/1.73m^2).
CI, confidence interval; CVEs, cardiovascular events; HR, hazard ratio.
†Per 10^5 person-years.

©2020 Wang Y-H et al. *JAMA Network Open*.

eTable 3. Association of Baseline (2010) Sleep Duration and First Cardiovascular Events or All-Cause Mortality

Outcome	Sleep duration in 2010, HR (95% CI)				
	**<6h n=4995**	**6-7h n=12275**	**7-8h n=11218**	**8-9h n=23320**	**≥9h n=737**
**First CVEs and all-cause mortality**					
Cases/person years	477/34036	927/83388	718/76349	2231/155982	65/4960
Incidence rate†	1401.4	1111.7	940.4	1430.3	1310.5
Age and sex adjusted	**1.27 (1.13-1.43)**	1.10 (0.99-1.21)	1.00 (Ref)	**1.34 (1.23-1.46)**	**1.40 (1.08-1.80)**
Multivariable adjusted*	**1.24 (1.10-1.39)**	1.08 (0.98-1.20)	1.00 (Ref)	**1.32 (1.21-1.44)**	**1.45 (1.13-1.87)**
**CVEs**					
Cases/person years	248/31685	541/83471	404/76462	1182/156139	30/4976
Incidence rate†	782.7	648.1	528.4	757.0	602.9
Age and sex adjusted	**1.25 (1.07-1.47)**	**1.17 (1.03-1.33)**	1.00 (Ref)	**1.33 (1.18-1.49)**	1.17 (0.81-1.70)
Multivariable adjusted*	**1.19 (1.01-1.40)**	**1.15 (1.01-1.31)**	1.00 (Ref)	**1.30 (1.15-1.41)**	1.17 (0.81-1.70)
**All cause mortality**					
Cases/person years	257/34377	463/84959	377/77594	1224/159382	39/5054
Moratality rate†	747.6	545.0	485.9	768.0	771.7
Age and sex adjusted	**1.22 (1.04-1.43)**	1.00 (0.87-1.15)	1.00 (Ref)	**1.30 (1.15-1.46)**	**1.53 (1.10-2.13)**
Multivariable adjusted*	**1.18 (1.01-1.38)**	1.00 (0.87-1.15)	1.00 (Ref)	**1.25 (1.10-1.41)**	**1.52 (1.09-2.11)**

*Adjusted for Age, sex, marital status (single, married, or divorced), occupation (Blue collar/white collar), average income (<500, 500-3,000, or ≥3,000 RMB/month), education (illiteracy or elementary, middle school, or college/university), physical activity (never, 1-2 times/week, or ≥3 times/week), smoking status (never, past, or current), drinking status (never, past, or current), salt intake (<6, 6-10, >10g/d), family history of stroke (yes/no), family history of MI (yes/no),hypertension (yes/no), Hyperlipidemia (yes/no), diabetes (yes/no), snoring frequency (never/rare, occasionally, or frequently), sleep duration in 2010, antihypertensive use (yes/no), hypoglycemic use (yes/no), lipid lowing agents use (yes/no), BMI (<18.5,18.5-25.0, 25.0-30.0, or ≥30.0 kg/m^2), fasting blood glucose (<4.0, 4.0-5.60, 5.60-6.10, 6.10-7.0, or ≥7.0 mmol/L), hs-CRP (<1.0, 1.0-3.0, 3.0-10.0, or ≥10.0 mg/L), SBP (<120, 120-140, ≥140mmHg), DBP (<80, 80-90, ≥90mmHg), eGFR (<30, 30-60, 60-90, or ≥90 ml/min/1.73m^2).
CI, confidence interval; CVEs, cardiovascular events; HR, hazard ratio.
†Per 10^5 person-years

©2020 Wang Y-H et al. *JAMA Network Open*.

eTable 4. Association Between Cumulative Average Sleep Duration During 2006 to 2010 and First Cardiovascular Events or All-Cause Mortality

Outcome	Average sleep duration during 2006-2010, HR (95% CI)				
	**<6h n=4018**	**6-7h n=13205**	**7-8h n=23793**	**8-9h n=11393**	**≥9h n=190**
**First CVEs and all-cause mortality**					
Cases/person years	381/26907	1110/89243	1933/160645	968/76679	26/1244
Incidence rate†	1416.0	1243.8	1203.3	1262.4	2090.0
Age and sex adjusted	1.02 (0.91-1.13)	0.98 (0.91-1.05)	1.00 (Ref)	1.07 (0.99-1.16)	**1.81 (1.23-2.67)**
Multivariable adjusted*	0.99 (0.88-1.11)	0.98 (0.91-1.06)	1.00 (Ref)	1.06 (0.98-1.15)	**1.75 (1.19-2.58)**
**CVEs**					
Cases/person years	209/25180	610/83799	1076/151163	501/72200	10/1168
Incidence rate†	830.0	727.9	711.8	693.9	856.2
Age and sex adjusted	1.09 (0.94-1.26)	0.98 (0.89-1.09)	1.00 (Ref)	1.02 (0.92-1.13)	1.08 (0.58-2.03)
Multivariable adjusted*	1.04 (0.89-1.21)	0.98 (0.88-1.08)	1.00 (Ref)	1.02 (0.92-1.14)	1.10 (0.59-2.06)
**All cause mortality**					
Cases/person years	202/27495	588/90951	1013/163602	540/78056	18/1264
Moratality rate†	734.7	646.5	619.2	691.8	1424.0
Age and sex adjusted	0.97 (0.84-1.13)	0.98 (0.89-1.09)	1.00 (Ref)	**1.12 (1.01-1.25)**	**2.41 (1.51-3.84)**
Multivariable adjusted*	0.98 (0.84-1.14)	0.99 (0.89-1.09)	1.00 (Ref)	**1.11 (1.00-1.23)**	**2.34 (1.46-3.72)**

*Adjusted for Age, sex, marital status (single, married, or divorced), occupation (Blue collar/white collar), average income (<500, 500-3,000, or ≥3,000 RMB/month), education (illiteracy or elementary, middle school, or college/university), physical activity (never, 1-2 times/week, or ≥3 times/week), smoking status (never, past, or current), drinking status (never, past, or current), salt intake (<6, 6-10, >10g/d), family history of stroke (yes/no), family history of MI (yes/no),hypertension (yes/no), Hyperlipidemia (yes/no), diabetes (yes/no), snoring frequency (never/rare, occasionally, or frequently), sleep duration in 2010, antihypertensive use (yes/no), hypoglycemic use (yes/no), lipid lowing agents use (yes/no), BMI (<18.5,18.5-25.0, 25.0-30.0, or ≥30.0 kg/m^2), fasting blood glucose (<4.0, 4.0-5.60, 5.60-6.10, 6.10-7.0, or ≥7.0 mmol/L), hs-CRP (<1.0, 1.0-3.0, 3.0-10.0, or ≥10.0 mg/L), SBP (<120, 120-140, ≥140mmHg), DBP (<80, 80-90, ≥90mmHg), eGFR (<30, 30-60, 60-90, or ≥90 ml/min/1.73m^2).
CI, confidence interval; CVEs, cardiovascular events; HR, hazard ratio.
†Per 10^5 person-years.

©2020 Wang Y-H et al. *JAMA Network Open*.
